# Prophylactic Treatment With Simvastatin Modulates the Immune Response and Increases Animal Survival Following Lethal Sepsis Infection

**DOI:** 10.3389/fimmu.2018.02137

**Published:** 2018-09-21

**Authors:** Jose A. F. Braga Filho, Afonso G. Abreu, Carlos E. P. Rios, Liana O. Trovão, Dimitri Luz F. Silva, Dalila N. Cysne, Johnny R. Nascimento, Thiare S. Fortes, Lucilene A. Silva, Rosane N. M. Guerra, Márcia C. G. Maciel, Carlos H. Serezani, Flávia R. F. Nascimento

**Affiliations:** ^1^Laboratory of Immunophysiology, Federal University of Maranhão, São Luís, Brazil; ^2^Programa de Pós-Graduação em Ciências da Saúde, Federal University of Maranhão, São Luís, Brazil; ^3^CEUMA University, São Luís, Brazil; ^4^Division of Infectious Diseases, Departments of Medicine, Pathology, Microbiology and Immunology, Institute for Infection, Immunology and Inflammation, Vanderbilt University School of Medicine, Nashville, TN, United States

**Keywords:** simvastatin, prophylactic treatment, sepsis, immunomodulation, macrophages, hydrogen peroxide, cytokines

## Abstract

Chronic use of statins may have anti-inflammatory action, promoting immunomodulation and survival in patients with sepsis. This study aimed to analyze the effects of pretreatment with simvastatin in lethal sepsis induced by cecal ligation and puncture (CLP). Male *Swiss* mice received prophylactic treatment with simvastatin or pyrogen-free water orally in a single daily dose for 30 days. After this period, the CLP was performed. Naïve and Sham groups were performed as non-infected controls. Animal survival was monitored for 60 h after the CLP. Half of mice were euthanized after 12 h to analyze colony-forming units (CFUs); hematological parameters; production of IL-10, IL-12, IL-6, TNF-α, IFN-γ, and MCP-1; cell counts on peritoneum, bronchoalveolar lavage (BAL), bone marrow, spleen, and mesenteric lymph node; immunephenotyping of T cells and antigen presenting cells and production of hydrogen peroxide (H_2_O_2_). Simvastatin induced an increase in survival and a decrease in the CFU count on peritoneum and on BAL cells number, especially lymphocytes. There was an increase in the platelets and lymphocytes number in the Simvastatin group when compared to the CLP group. Simvastatin induced a greater activation and proliferation of CD4+ T cells, as well as an increase in IL-6 and MCP-1 production, in chemotaxis to the peritoneum and in H_2_O_2_ secretion at this site. These data suggest that simvastatin has an impact on the survival of animals, as well as immunomodulatory effects in sepsis induced by CLP in mice.

## Introduction

Sepsis is defined as a complex systemic inflammatory response caused by an uncontrolled infection with activation of both pro- and anti-inflammatory responses. The development and progression of sepsis are multifactorial, affecting the cardiovascular, neuronal, endocrine, and immune systems (1, 2). It is the second most frequent cause of death among patients hospitalized in UTIs and the 10th most frequent cause of mortality in general (3).

Clinical manifestations of sepsis such as fever, hypercoagulation, and peripheral hypotension are derived from the release of inflammatory cytokines, such as IL-1β, IL-6, IL-17, TNF-α, and anti-inflammatory cytokines: IL-4, IL-10, IL-13, TGF-β (4).

Severe sepsis and septic shock are the leading causes of morbidity and mortality in hospitalized patients and immunosuppressed individuals (5). Usually, patients are treated with antimicrobials that can cause cardiovascular and renal changes, with serious side effects (6). In addition, the worsening of the condition is very rapid, and there is no adequate therapeutic response. For these reasons, developing new treatment strategies for these patients has become necessary.

In this context, statins emerge as a potential therapy, since it was shown that patients who used statins were less likely to develop sepsis (7). Statins are medications widely used to treat hypercholesterolemia by reducing the serum concentration of low-density lipoprotein (LDL) cholesterol. In enzymatic terms, statins act by inhibiting 3-hydroxy-3-methylglutaryl-coenzyme A reductase (HMG-CoA reductase) preventing the conversion of HMG-CoA to mevalonic acid, which is a cholesterol precursor (8).

The use of statins reduces the expression of adhesion molecules, both in monocytes and neutrophils, and in endothelial cells, with a consequent decrease in the migration of polymorphonuclear leukocytes to tissues (9). In addition, they decrease the synthesis of acute-phase proteins, such as C-reactive protein, which is used as an inflammatory and cardiovascular risk marker, and reduce pro-cytokine secretion of inflammatory agents, such as IL-1β and IL-6, without TNF-α influences (9, 10).

Niessner et al. (11) reported that statins play a key role in modulating leukocyte and monocyte functions, reduce oxidative stress, and improve endothelial function and platelet activity. Simvastatin also reduced the systemic response to endotoxin administration and decreased the expression of Toll-like receptors, which play a key role in sepsis.

Another important factor in the inflammatory context is that statins increase the expression of endothelial nitric oxide synthase (eNOS), which is essential for adequate endothelial function. This may be instrumental in restoring the balance between eNOS and inducible nitric oxide synthase (iNOS), which is disrupted in sepsis, and is an important factor in the genesis of septic shock (9). In sepsis experiments, simvastatin proved to be effective in hemodynamic stabilization in mice with sepsis induced by cecal ligature and puncture (CLP). In addition to stabilizing hemodynamics, these animals showed improved responses to beta-adrenergic vasopressin drugs (dobutamine), significantly increasing their blood pressure, with decreased polymorphonuclear cell adhesion to the previously activated endothelium (12).

Despite this evidence, little is known about the immunological mechanisms involved in this apparent protective effect of simvastatin in the context of sepsis, considering both lymphocyte and macrophage activation, as well as about its possible effects on the inflammatory process and on the various metabolic and organic dysfunctions accompanying sepsis, which led to the present study.

## Materials and methods

### Animals and treatment

In this study, 12-week-old male Swiss mice were used. The animals were obtained from the Central animal house of the Federal University of Maranhão (UFMA) and were maintained with water and food *ad libitum*. All procedures were evaluated and approved by the Research Ethics Committee of the Federal University of Maranhão (Protocol: 012975/2008-43).

The animals were separated into 4 groups (10 animals/group). In the control group (CLP group), 200 μL of apyrogenic water was administered orally in a single daily dose. In the Simvastatin group, 200 μL of simvastatin (40 mg/kg) was administered orally in a single daily dose. The treatments lasted 30 days and the dosage used was chosen according to Winkler et al. (13). Two additional groups were performed as negative controls, the Naïve group, which received no procedures and the Sham group, which was operated but had no perforations in the cecum.

### Cecal ligature and puncture (CLP)

After 30 days of pretreatment with simvastatin or water, the animals underwent CLP. Polymicrobial sepsis was induced using the CLP method described by Benjamim et al. (14), with modifications in the anesthetic used. Initially, the mice were anesthetized with 25 mg/kg ketamine hydrochloride and 20 mg/kg xylazine hydrochloride according to Machado et al. (15). Then, a laparotomy was performed, and the cecum was mobilized, ligated below the cecal valve, and punctured 10 times with an 18-gauge needle to induce lethal sepsis. The cecum was placed back into the peritoneal cavity, and the abdomen was closed in two layers. Saline (0.5 mL/10 g body weight) was given subcutaneously to CLP animals for fluid resuscitation. After 12 h of CLP, half of the mice were euthanized with an overdose of anesthetic (150 mg/kg ketamine hydrochloride and 120 mg/kg xylazine hydrochloride) and another half of the animals were maintained alive to evaluate the lifespan (*n* = 5). The mortality of the animals was recorded every 12 h. The Sham group was submitted to all the procedures with exception of the perforations.

### Evaluation of hematological parameters

To determine the hematological parameters, 100 μL of blood was collected by retrorbital route, 12 h after the CLP. Blood was stored in 1.5 mL tubes with ethylenediaminetetraacetic acid (EDTA) as an anticoagulant. An automated hematology analyzer (Poch-100iV Diff, Sysmex Corp) was used. The following parameters were analyzed: red blood cells, hemoglobin, hematocrit, mean corpuscular volume (MCV), mean corpuscular hemoglobin (MCH), mean corpuscular hemoglobin concentration (MCHC), red-cell distribution width (RDW), and number of total leukocytes, neutrophils, lymphocytes, monocytes, and platelets.

### Bronchoalveolar lavage

The trachea of the animals was exposed, fitted with a cannula, and 1 mL of cold PBS was injected in the bronchoalveolar space using a syringe. After a short massage on the chest, the solution was aspirated at least three times. To determine the total number of cells in the bronchoalveolar lavage, cell suspensions were stained with crystal violet (0.05%) in 30% acetic acid at a ratio of 9:1. Cells were counted in a Neubauer chamber using an optical microscope (×400). For differential counting, slides were prepared using a cytospin (800 rpm/3 min) and then were fixed and stained using an Instant-Prov Kit (Newprov, Pinhais, Brazil).

### Colony-forming unit (CFU) determination

The mice were killed 12 h after the CLP. The skin of the abdomen was cut open on the midline after thorough disinfection and without injury to the muscle, and the peritoneal cavity was washed with 2 mL of sterile phosphate buffered solution (PBS). Aliquots of serial log dilutions of the obtained peritoneal fluid were plated on Mueller-Hinton agar dishes (Difco Laboratories, Detroit), colony-forming units (CFU) were counted after overnight incubation at 37°C, and the results were expressed as the number of CFU per peritoneal cavity.

### Collection and counting of cells from the peritoneal lavage and lymphoid organs

The animal's peritoneal cavity was washed with 5 mL sterile PBS. After abdominal wall excision, cell suspensions were obtained by aspiration using a syringe and needle, transferred to conical-bottom polypropylene tubes, and maintained in an ice bath at 4°C until the cells were counted. After collection of the peritoneal lavage, the spleen, and mesenteric lymph nodes were collected, weighed, and crushed. The femur was perfused with 1 mL PBS to obtain bone marrow cells. For total cell number counting, 90 μL of each cell suspension was fixed and stained with 10 μL 0.05% crystal violet in 30% acetic acid. The cells were counted using a Neubauer chamber with the aid of an optical microscope at 400× magnification (16). Differential peritoneal cell counts were determined using the cytospin system (800 rpm/3 min), fixed, and stained with the Instant-Prov kit (Newprov, Pinhais, Brazil). The percentage of cell subpopulations was calculated based on the count of 100 cells and transformed in absolute number based on the total count.

### Phenotype characterization of leukocytes

The phenotypes of cells from the mesenteric lymph node were characterized using commercial monoclonal antibodies (BD Biosciences, San Jose, CA), according to the manufacturer's instructions. Two panels of antibodies were used, one for adherent cells, including anti-CD14 (FITC) and anti-IA/IE (PE), and the other for non-adherent cells, anti-CD3 (FITC), anti-CD4 (PE), and anti-CD8 (PerCP). After the acquisition of 10,000 events in a FACSCalibur flow cytometer, the obtained data were analyzed using FlowJo software.

### Quantification of cytokines

The cytometric bead array (CBA) technique was used for the quantification of TNF-α, MCP-1, IL-6, IL-10, IL-12, and IFN-γ in serum, as described by Maciel et al. (17), using the mouse inflammation cytokine kit (Becton Dickinson Biosciences, San Jose, CA, EUA).

### Determination of hydrogen peroxide release (H_2_O_2_)

To evaluate H_2_O_2_ release, a horseradish peroxidase-dependent phenol red oxidation microassay was used (18, 19). In this assay, two million peritoneal cells were suspended in 1 mL freshly prepared phenol red solution that consisted of ice-cold Dulbecco's PBS containing 5.5 mM dextrose, 0.56 mM phenol red (Sigma), and 8.5 U/mL horseradish peroxidase type II (Sigma). One hundred microliters of the cell suspension was added to each well and incubated in the presence or absence of 10 ng phorbol myristate acetate (PMA) (Sigma), for 1 h at 37°C in a humid atmosphere containing 5% CO_2_ and 95% air. The plates were centrifuged once at 150 × g for 3 min and the supernatants were collected and transferred to another plate. The reaction was stopped with 10 μL 1N NaOH. The absorbance was measured at 620 nm with a microplate reader (MR 5000, Dynatech Laboratories Inc., Gainesville, VA, USA). Conversion of absorbance to μM H_2_O_2_ was done by comparison to a standard curve obtained with known concentrations of H_2_O_2_ (5–40 μM).

### Statistical analysis

Results were expressed as the mean ± standard deviation. The normality of data was evaluated by the D'Agostino-Pearson test. Statistical analysis was performed using ANOVA followed by Student's *t*-test for parametric data and the Mann-Whitney for non-parametric data, using Graph Pad Prism software, version 6.0. The differences were considered to be significant when *p* ≤ 0.05. The lifespan of the mice was demonstrated using the Kaplan-Meier curve, and the log-rank statistical test was applied to compare the curves. All experiments were repeated at least two times.

## Results

### Prophylactic treatment with simvastatin increases the survival of animals with sepsis

**Figure 1:**
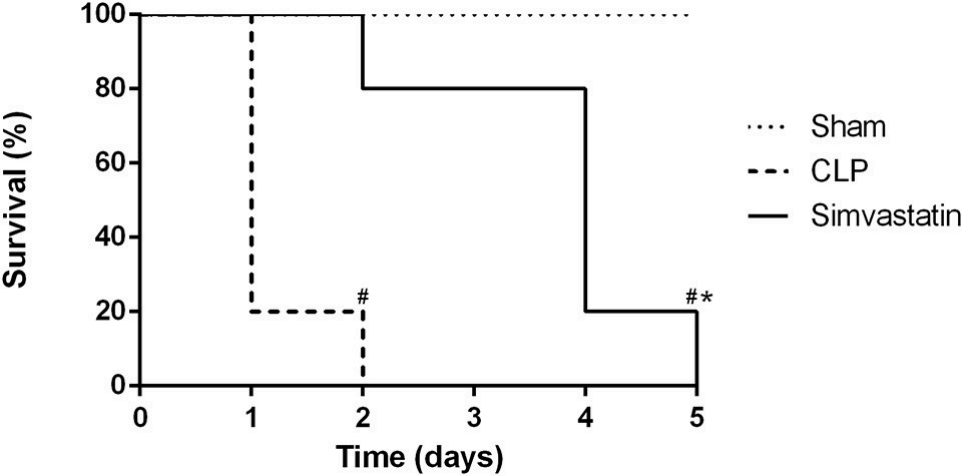
Survival curve of animals with sepsis. Animals received water (CLP group) or simvastatin (Simvastatin group) orally for 30 days when CLP was induced. Each group consisted of 5 animals. The groups were clinically evaluated for the number of deaths after 12 h of CLP at regular 12 h intervals for 60 h. The Sham group was submitted to all the procedures with exception of cecum perforation. *p < 0.05 compared to CLP group. ^#^*p* < 0.05 when compared to the Sham group.

Control animals (CLP group) died within 48 h. However, it was only after 30 h that the first death occurred in the simvastatin group. In this group, over 80% of the animals survived up to the 50 h follow-up. The last death of the simvastatin group occurred at 60 h (Figure 1). The animals from the Sham group remained alive until the last day of observation.

### Simvastatin induces a reduction in the number of CFU at the focus of the infection

**Figure 2:**
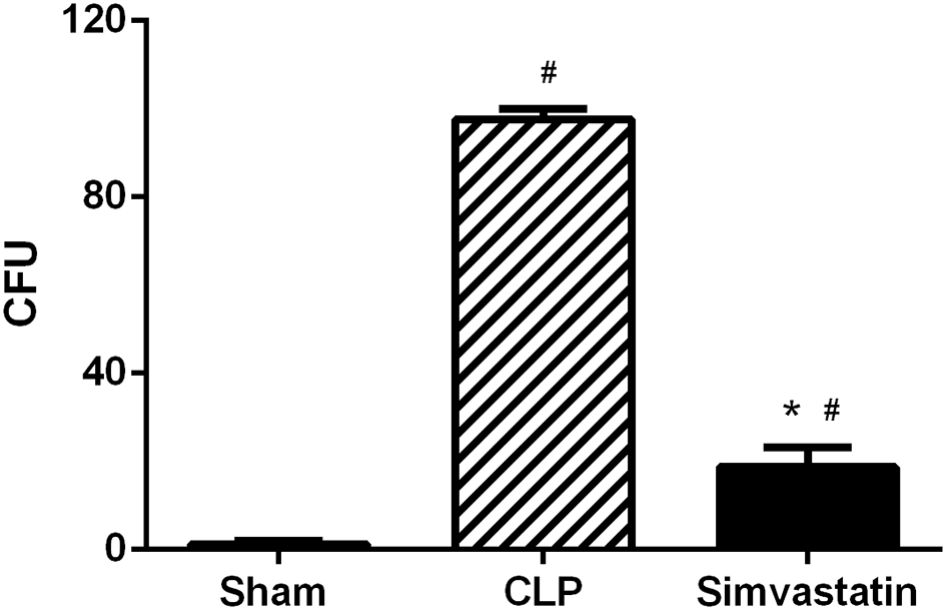
Effect of pretreatment with simvastatin on the number of CFU in peritoneum. Animals received water (CLP group) or simvastatin (Simvastatin group) orally for 30 days when CLP was induced. Each group consisted of 5 animals. After 12 h of CLP, aliquots of the peritoneal lavage were diluted and plated on Mueller-Hinton agar and incubated at 37°C for 18 h for counting of CFU. The data are represents as mean ± S.D. of CFU × 10^4^. The Naïve group received no procedures and the Sham group was submitted to all the procedures with exception of cecum perforation. *p < 0.05 compared to CLP group. ^#^*p* < 0.05 when compared to the Sham group.

Pretreatment with simvastatin induced a reduction in the bacterial counts in the peritoneum, the focus of the infection when compared to the CLP group (Figure 2). At the Sham group no CFU was detected in the peritoneum.

### Simvastatin inhibits the recruitment of cells into the bronchoalveolar space

**Figure 3:**
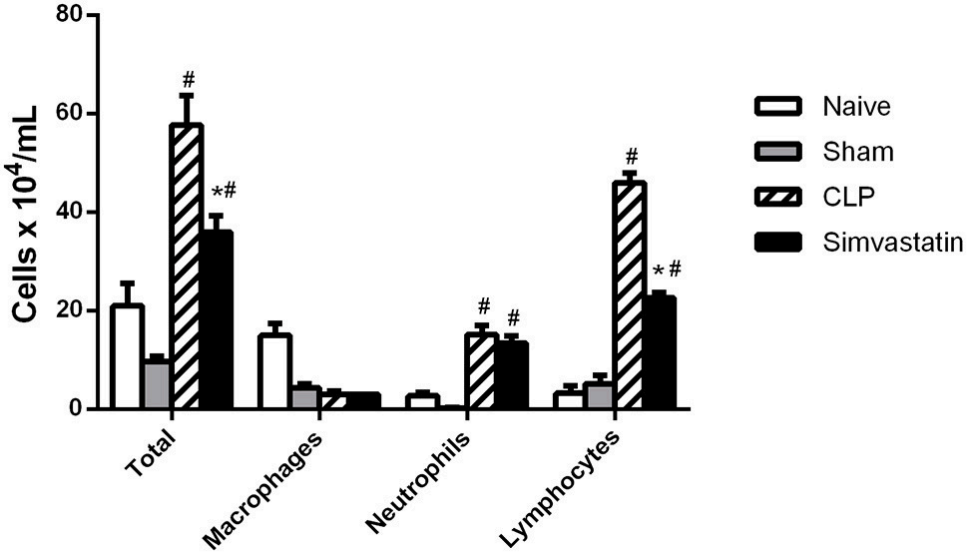
Effect of pretreatment with simvastatin on the total and differential counts of Bronchoalveolar lavage cells (BAL). Animals received water (CLP group) or simvastatin (Simvastatin group) orally for 30 days when CLP was induced. The total and differential count of the bronchoalveolar cells was made 12 h after the induction of sepsis by the CLP model. The results represent the mean ± S.D. of 5 animals per group. The Naïve group received no procedures and the Sham group was submitted to all the procedures with exception of cecum perforation. *p < 0.05 compared to the CLP group. ^#^*p* < 0.05 when compared to the Naïve and Sham groups.

Considering that pulmonary inflammation is one of the causes of death in sepsis, we investigated the leukocyte distribution in bronchoalveolar lavage. CLP and Simvastatin groups showed increased recruitment of inflammatory cells, especially neutrophils and lymphocytes, to the BAL when compared to Naïve and Sham groups. However, the treatment with simvastatin reduced this leukocyte infiltrate, specially the number of lymphocytes when compared to CLP group. On the other hand, Simvastatin did not affect the migration of macrophages and neutrophils when compared to CLP group (Figure 3).

### Simvastatin induces increased lymphocytes and platelets in the blood

No significant differences between Naïve and Sham groups were observed in blood analysis. However, CLP and Simvastatin groups showed a significant decrease in the number of platelets and an increase in the number of leukocytes, specially lymphocytes, when compared to Naïve and Sham groups. The number of platelets in the Simvastatin group was increased when compared to the CLP group, but it was still smaller than the Sham group. The treatment with simvastatin also increased the number of lymphocytes in the blood when compared to the CLP group (Table 1).

**Table 1:** The effect of oral use of simvastatin in hematological parameters in septic mice.

**Hematological parameters**	**Naive**	**Sham**	**CLP**	**Simvastatin**
Hematocrit (%)	50.8 ± 0.5	46.9 ± 1	46.2 ± 0.9^a^	45.3 ± 1.1
Hemoglobin (g/dL)	15.0 ± 0.2	15.1 ± 0.4	13.8 ± 0.2	13.8 ± 0.5
Erythrocytes (×10^6^/dL)	10.0 ± 0.3	10.9 ± 0.2	9.1 ± 0.2	9.5 ± 0.4
Platelets (×10^3^/dL)	1286.0 ± 218.7	1650.0 ± 397.3	556.2 ± 52.5^#^	832.8 ± 77.5^#^^*^
Leukocytes (×10^3^/dL)	3.1 ± 0.2	2.2 ± 0.29	8.4 ± 0.6^#^	8.0 ± 0.9^#^
Neutrophils (×10^3^/dL)	1.8 ± 0.1	1.5 ± 0.3	3.3 ± 0.6^#^	3.0 ± 0.5^#^
Lymphocytes (×10^3^/dL)	1.2 ± 0.2	0.8 ± 0.2	4.9 ± 0.2^#^	5.6 ± 0.4^#^^*^
Monocytes (×10^3^/dL)	0.05 ± 0.02	0.1 ± 0.02	0.1 ± 0.0	0.2 ± 0.1

a The results are presented as the mean ± SEM;

* p < 0.05 when compared to the CLP group;

# *p < 0.05 when compared to the Naïve and Sham groups*.

### Effects of pretreatment with simvastatin on the total cell count of bone marrow, spleen, and mesenteric lymph node

**Figure 4:**
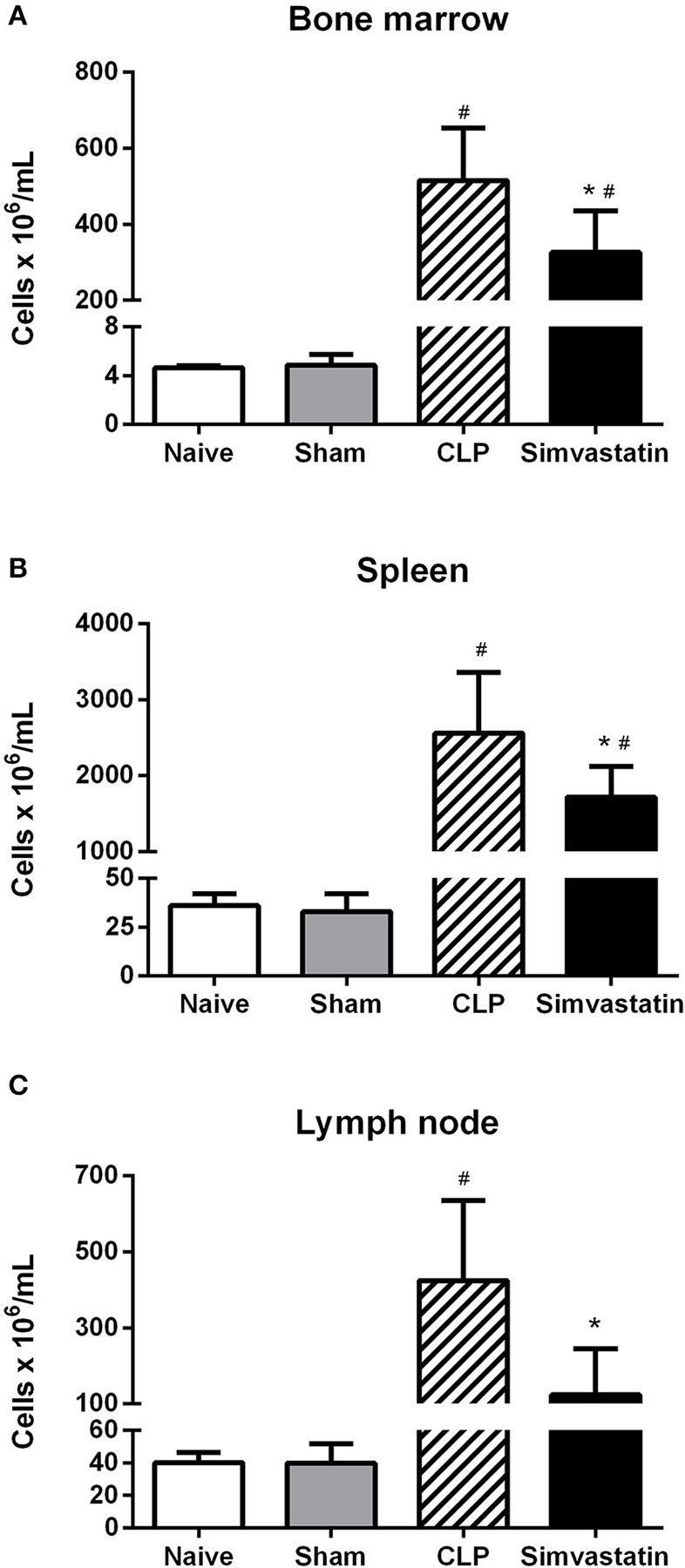
Effect of pretreatment with simvastatin on the total cell count of bone marrow, spleen, and lymph node in septic mice. The animals received water or simvastatin orally for 30 days when CLP was induced. After 12 h of CLP, the bone marrow **(A)**, spleen **(B)**, and lymph node **(C)** were collected and quantified. The results represent the mean ± S.D. of 5 animals per group. The Naïve group received no procedures and the Sham group was submitted to all the procedures with exception of cecum perforation. *p < 0.05 compared to control group. ^#^p < 0.05 when compared to the Naïve and Sham groups.

Bone marrow, spleen and mesenteric lymph node cells were also collected to investigate whether simvastatin could induce any changes in lymphoid cells. The results from Simvastatin and CLP groups were similar and always high than those counts observed in the Naïve and Sham groups (Figure 4).

### Pretreatment with simvastatin induces an increase in the percentage of helper T lymphocytes and the expression of class II MHC in animals with sepsis

**Figure 5:**
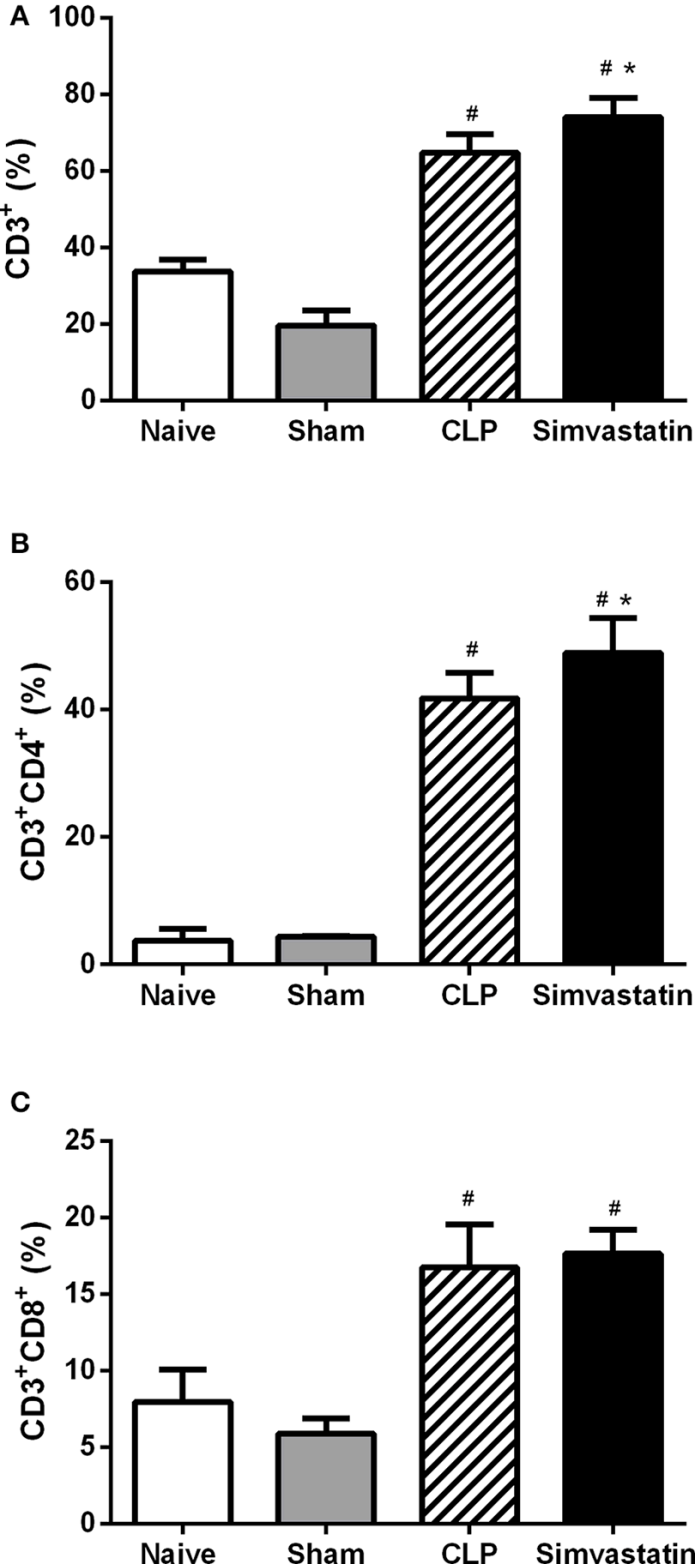
Effect of simvastatin pretreatment on T cells populations in the lymph node. Animals received water (CLP group) or simvastatin (Simvastatin group) orally for 30 days when CLP was induced. Immunophenotyping was performed 12 h after the induction of lethal sepsis by the CLP model. **(A)** % of T lymphocytes; **(B)** % of T-helper lymphocytes; **(C)** % of T-cytotoxic lymphocytes. The results are expressed as percentage values. These values represent the mean ± S.D. of 5 animals per group. The Naïve group received no procedures and the Sham group was submitted to all the procedures with exception of cecum perforation. *p < 0.05 compared to the CLP group. ^#^*p* < 0.05 when compared to the Naïve and Sham groups.

Control group showed an increased percentage of T lymphocytes when compared to Naïve and Sham groups. Treatment with simvastatin increased the percentage of both total T lymphocytes (Figure 5A) and helper T lymphocytes (CD3+CD4+) (Figure 5B) but did not affect the percentage of cytotoxic T lymphocytes (CD3+CD8+) when compared to Control group (Figure 5C).

**Figure 6:**
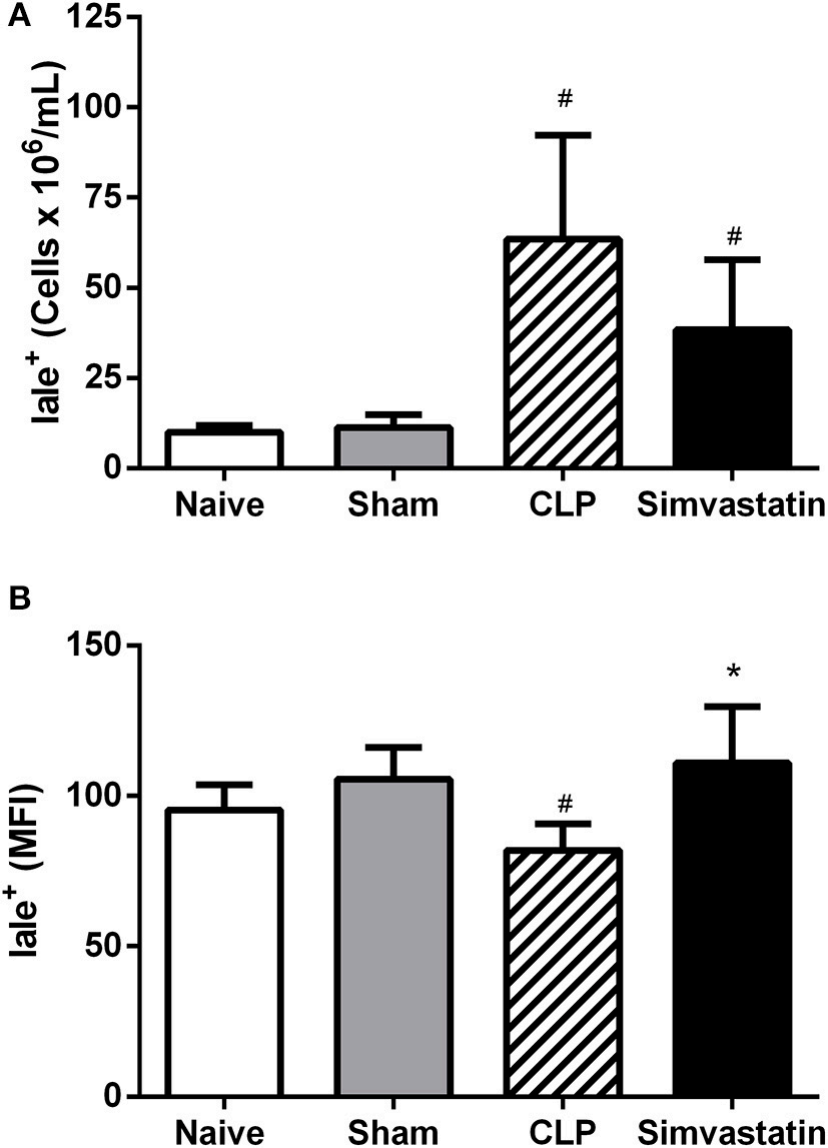
Effect of simvastatin pretreatment on the number of antigen presenting cells in the lymph node. Animals received water (CLP group) or simvastatin (Simvastatin group) orally for 30 days when CLP was induced. Immunophenotyping was performed 12 h after the induction of lethal sepsis by the CLP model. **(A)** % of antigen presenting cells (Ia-Ie+); **(B)** The mean fluorescence intensity (MIF). These values represent the mean ± S.D. of 5 animals per group. The Naïve group received no procedures and the Sham group was submitted to all the procedures with exception of cecum perforation. *p < 0.05 compared to the CLP group. ^#^*p* < 0.05 when compared to the Naïve and Sham groups.

The presence of antigen-presenting cells (APCs) was also investigated by the expression of class II MHC molecules (Ia-Ie marker). The CLP group showed an increase in the number of APCs (IaIe+) and a decrease of Class II MHC expression (MFI) when compared to Naïve and Sham groups (Figures 6A,B). Simvastatin treatment did not change the number of APCs (IaIe+), however there was an increased expression of class II MHC molecules in APC from this group (Figure 6B) when compared to the CLP group.

### Pretreatment with simvastatin induces an increase in inflammatory cytokines in animals with sepsis

**Figure 7:**
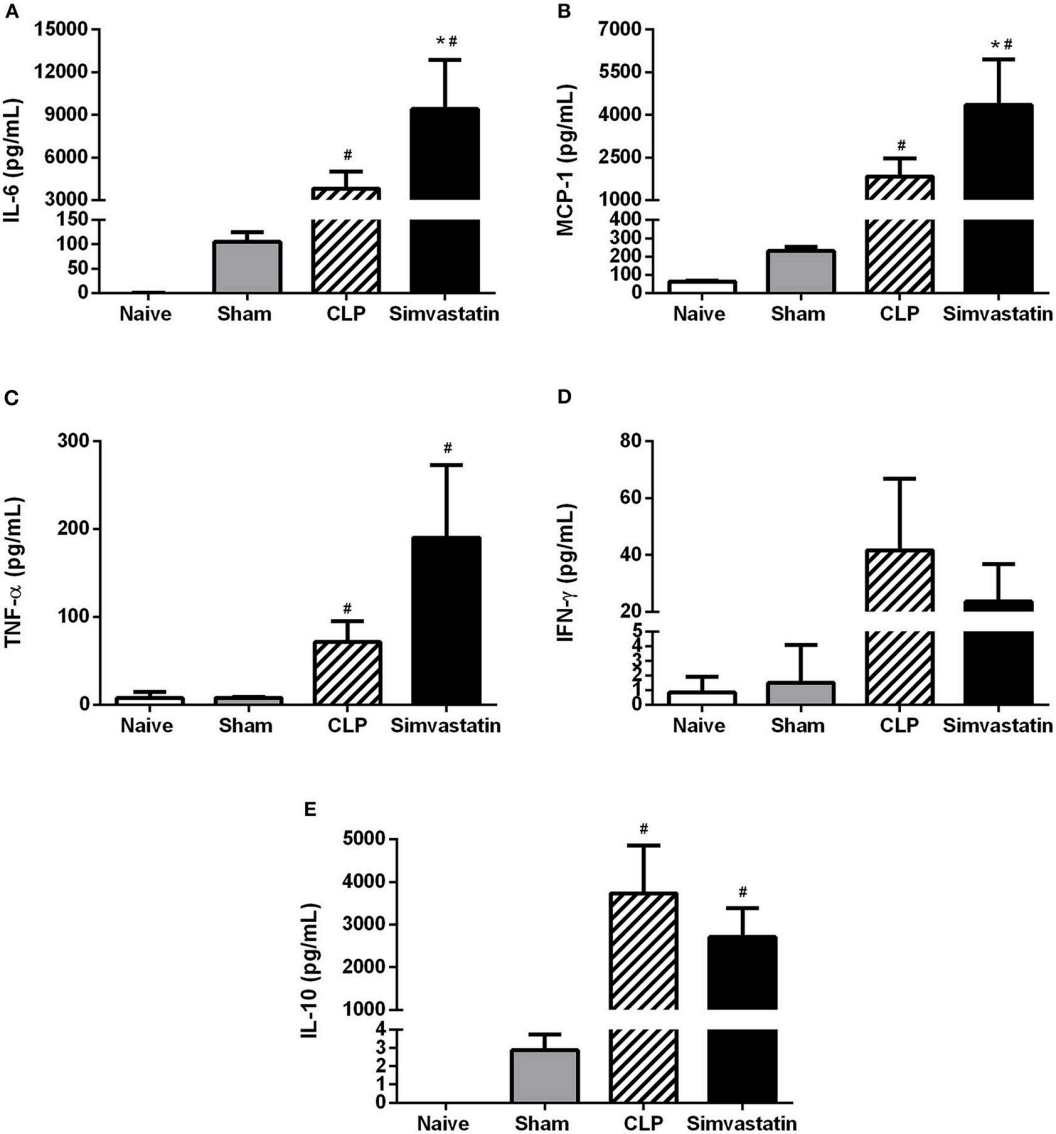
Effect of simvastatin pretreatment on serum cytokines. Animals received water (CLP group) or simvastatin (Simvastatin group) orally for 30 days when CLP was induced. IL-6 **(A)**, MCP-1 **(B)**, TNF-α **(C)**, IFN-γ **(D)**, and IL-10 **(E)** dosing was done 12 h after the induction of lethal sepsis by the CLP model. These values represent the mean ± S.D. of 5 animals per group. The Naïve group received no procedures and the Sham group was submitted to all the procedures with exception of cecum perforation. *p < 0.05 compared to the control group. ^#^*p* < 0.05 when compared to the Naïve and Sham groups.

The CLP induced an increase of all the cytokines when compared to Naïve and Sham groups. Simvastatin treatment increased IL-6 and MCP-1 production (Figures 7A,B), without significant differences in TNF-α, IFN-γ, and IL-10 expression (Figures 7C–E) when compared to CLP group. The IL-12 was not detected.

### Effect of pretreatment with simvastatin on the phenotypic and functional characteristics of peritoneal cells

**Figure 8:**
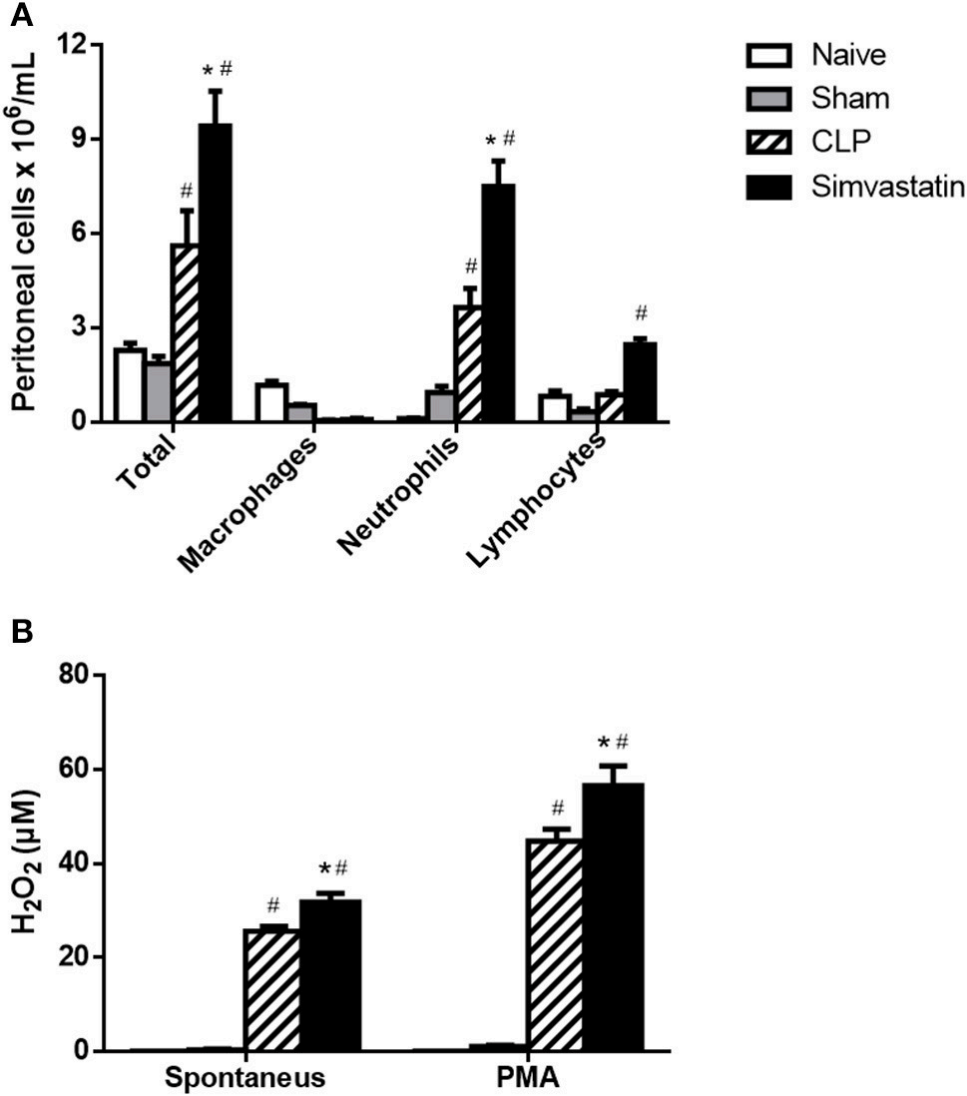
Effect of simvastatin pretreatment on the recruitment and activation of peritoneal cells. Animals received water (CLP group) or simvastatin (Simvastatin group) orally for 30 days when CLP was induced. **(A)** Total and differential cell counts were made 12 h after the CLP. **(B)** Release of H_2_O_2_, spontaneous and induced by PMA, into the peritoneum of animals with sepsis induced by CLP. The results represent the mean ± S.D. of 5 animals per group. The Naïve group received no procedures and the Sham group was submitted to all the procedures with exception of cecum perforation. *p < 0.05 compared to the CLP group. ^#^*p* < 0.05 when compared to the Naïve and Sham groups.

In the peritoneal cavity, CLP induced an increase of inflammatory cell influx when compared to Naïve and Sham groups. Moreover, the total cell count was significantly increased in the Simvastatin group (Figure 8A). Analyzing the cell populations, there were an increased number of neutrophils and lymphocytes compared to the controls, with no difference in the number of macrophages (Figure 8A).

The activation of the peritoneal cells was evaluated by the production of NO and hydrogen peroxide. The NO production was not detected neither in the peritoneum not in the lymph node cells cultures. However, there was an increase of both spontaneous and PMA-stimulated H_2_O_2_ release in by peritoneal cells from Simvastatin group when compared to CLP group (Figure 8B). The Naïve and Sham groups had no production of NO and H_2_O_2_.

## Discussion

In this work, it has been demonstrated that the use of simvastatin for 30 days protects animals from lethal sepsis and increases the survival of animals. This effect is related to the decrease of CFUs in the infectious focus and pulmonary infiltrate, inhibition of peripheral thrombocytopenia, increase in the percentage of helper T lymphocytes and expression of MHC class II molecules in macrophages, as well as of microbicidal mediators produced by the phagocytic cells.

Considering that the animals from both groups developed clinical signs of sepsis, the great benefit that chronic lipid-lowering therapy had on the animals was evident, since the animals from the simvastatin group died later than those in the control group. These results are in agreement with Ando et al. (20), who used animals with sepsis induced by intraperitoneal injection of lipopolysaccharide (LPS) and demonstrated an increase in survival in mice treated with cerivastatin compared to the control group. In another study, Ajrouche et al. (3) compared 105 patients who had been treated with statins with 246 patients who did not use lipid-lowering drugs and showed a decrease in mortality and a reduction in C-reactive protein levels. Similarly, Ou et al. (21) observed that patients who chronically used high-potency statins (rosuvastatin 10 mg, atorvastatin 20 mg, and simvastatin 40 mg) had an additional survival benefit when compared to chronic low-dose statins and, especially, when compared to the control group.

The increase in survival observed in the Simvastatin group may be associated with a decrease in the number of CFUs at the focus of the infection (peritoneum), which could be related to a microbicidal effect of the drug used or to its effect stimulating host microbicidal mechanisms. However, the possibility of an antimicrobial effect of statin, as described previously (22, 23), can be disproven since there were no differences in CFU number in blood cultures and BAL (data not shown), suggesting that its effectiveness could be due to the immune activation at the site of infection.

In infection, resident macrophages are able to phagocytose microorganisms and to secrete IL-1 and TNF-α. These cytokines, in turn, stimulate the recruitment of neutrophils, monocytes and T lymphocytes to the site of infection. This process is mediated by selectins, integrins, and chemokines (10, 24). In fact, simvastatin induced an increase in the number of cells recruited to the infectious focus, especially related to neutrophils and lymphocytes. However, the opposing effect was observed at the lung, since occurred a decreased infiltration of inflammatory cells in the BAL in the simvastatin group, with a smaller number of lymphocytes. These data are in agreement with Merx et al. (12), who demonstrated a reduction in leukocyte chemotaxis in inflammatory processes after the use of statins. These authors collected mononuclear cells from animals treated with simvastatin or untreated controls, and submitted them to cystine pre-stimulated endothelial cell adhesion assays. After 5 min and under physiological conditions of flow, the results showed a reduced amount of monocytes in the CLP+ simvastatin group adhered to the endothelial surface when compared to the control. Likewise, Almog showed decreased expression of selectins and integrin ligands in endothelial cells in the presence of statins (9). It is important to note that pulmonary inflammation is a frequent lethal complication of sepsis. Thus, a reduction of this cellular infiltrate at this site is a beneficial effect of simvastatin and could, at least partially, explain the increase of survival.

Despite the decrease in cellular recruitment to the bronchoalveolar space, simvastatin induced an increase in the percentage of CD4+ T lymphocytes in the lymph node, suggesting a greater proliferation and activation of these cells, what could justify the increase of lymphocytes in the blood. This increase may be also related to the increased expression of class II MHC molecules in the host cells, since the presentation of antigens via this molecule is a condition for the activation and proliferation of helper T cells. This result partially disagrees with the studies of Sun and Fernandes (25) and Ghittoni et al. (24) that demonstrated that statins have the ability to decrease the expression of class II MHC molecules on the cell surface of antigen-presenting cells, as well as to decrease the migration of these cells to the sentinel lymph node.

In addition to the increased expression of MHC class II molecules, there was a significant increase in the serum concentration of IL-6 and MCP-1, without significant differences in IL-10, IFN-γ, and TNF-α. Sun and Fernandes (25) already showed an increase in the mRNA expression for IL-6 and MCP-1 induced by lovastatin in LPS-stimulated dendritic cells. These results suggest that the effect of simvastatin on the increase of survival is not related to its anti-inflammatory activity, but to a modulation of immune response directed to antimicrobial action.

Finally, to investigate whether a greater cellular activation occurred at the focus of the infection that justified the reduction of the CFUs and consequent increase in the survival of the animals, the recruitment of cells to the peritoneal cavity and the microbicidal capacity of these cells by the production of H_2_O_2_. The animals from the simvastatin group presented significantly increased values of H_2_O_2_ in the peritoneum, the focus of the CLP infection. Macrophages and neutrophils are known to contain enzyme-rich cytoplasmic granules, one of which is NADPH oxidase or phagocyte oxidase. This converts molecular oxygen into superoxide anions, free radicals of oxygen and hydrogen peroxide; all are microbicidal agents in both phagolysosomes and extracellular microorganisms (26). In this study, it was observed that the peritoneal phagocytes from the animals of the simvastatin group secreted an increased amount of hydrogen peroxide compared to the control animals. This difference became more important when such cells were stimulated by PMA. The microbicidal potential of these cells is even more evident when the smaller number of cells that migrate to the peritoneum as a function of simvastatin are observed, i.e., fewer cells migrating to the peritoneum, but with a greater microbicidal capacity. This fact, together with a decreased number of CFUs in the peritoneum and an increased number of inflammatory cells in the peritoneal lavage, show that this statin is able to exert immunomodulatory effects on the peritoneum.

Altogether, the data indicate that pretreatment with simvastatin was able to increase the survival of animals with sepsis induced by CLP, because of its ability of improve the immune response, specially the microbicidal activity, the macrophage ability to present antigens to T cells and also, the immunomodulatory properties related to inflammatory cytokines. Considering the increase of class II MHC molecules expression, hydrogen peroxide secretion and inflammatory cytokines, is reasonable to suppose that the simvastatin could be directing the macrophages to a M1 polarization in the time evaluated in this model. Moreover, it is important to mention that immunomodulatory effect of simvastatin cannot be considered the only mechanism associated to the increase of survival since Merx et al. (12) have demonstrated that the improvement in hemodynamic functions played a major role in the survival benefit of simvastatin pre-treatment in mice with sepsis.

It is important to emphasize that simvastatin is widely used to control hypercholesterolemia and hypertriglyceridemia, and since its pharmacokinetics and pharmacodynamics are already widely known, the use of this drug as an adjuvant in the treatment of sepsis could be a good alternative to control the manifestations associated with sepsis and change the poor prognosis of sepsis and its related conditions.

## Ethics statement

All performed procedures were authorized by the National Council for Animal Control and Experimentation (CONCEA) and approved by the Animal Use Ethics Committee of the UFMA under protocol number 23115. 012975/2008-43.

## Author contributions

JB, CR, TF, MM, and FN conceived and designed the experiments. JB, CR, TF, LT, DS, DC, and JN performed the experiments. JB, AA, DC, JN, LS, and FN analyzed the data. FN and CS contributed reagents, materials, and analysis tools. JB, AA, MM, LS, RG, CS, and FN wrote the paper.

### Conflict of interest statement

The authors declare that the research was conducted in the absence of any commercial or financial relationships that could be construed as a potential conflict of interest.
